# A Case of Mirtazapine-Induced Pancreatitis

**DOI:** 10.7759/cureus.37129

**Published:** 2023-04-04

**Authors:** Hunain Aslam, Khalid Ahmed, Peter A Iskander, Mark M Aloysius, Vikas Khurana, Simin Nasr, Mohammad Asim Amjad

**Affiliations:** 1 Internal Medicine, The Wright Center for Graduate Medical Education, Scranton, USA; 2 Gastroenterology, The Wright Center for Graduate Medical Education, Scranton, USA; 3 Family Medicine, The Wright Center for Graduate Medical Education, Scranton, USA

**Keywords:** peri-pancreatic inflammation, inflammation, plasmapheresis, acute pancreatitis, antidepressants, mirtazapine

## Abstract

Acute pancreatitis is a concerning cause of hospitalization in the United States, with the most common etiologies being secondary to alcohol abuse and gallstones. Rarely, medications can trigger this inflammatory response, whether via direct toxic effects or other metabolic derangements. Mirtazapine is an antidepressant that has been associated with elevations in triglyceride levels on initiation. Relatedly, high triglyceride levels and autoimmune disorders are other causes of pancreatitis exacerbations. Here, we present the case of a female who was started on mirtazapine therapy and found to have elevated triglyceride levels. The course was complicated by acute pancreatitis requiring plasmapheresis, despite medication discontinuation, to which she responded well.

## Introduction

Pancreatitis is an inflammatory condition resulting from a pancreatic parenchymal injury. Medications account for up to 2% of acute pancreatitis (AP) cases [1]. The World Health Organization (WHO) database lists numerous medications associated with AP [2]. Antidepressants are infrequently associated with AP, and no specific class of antidepressants is notably linked [3]. A few case reports in the literature, however, suggest an association between Mirtazapine and AP, potentially due to mirtazapine-induced hypertriglyceridemia. Here, we present the case of a patient with hypertriglyceridemia likely secondary to mirtazapine who further developed AP.

## Case presentation

A 33-year-old female with a medical history of anxiety, depression, migraine headaches, and seasonal allergies presented to the hospital with complaints of intractable nausea and acute abdominal pain. She denied any personal/family history of gallbladder disease, nor was there any documented history of errors in lipid metabolism. She endorsed occasional alcohol consumption, and her surgical history was inclusive of an appendectomy, cesarean section, and tubal ligation several years prior.

Initially, the patient described the abdominal pain as vague and dull, rated three out of ten in severity, and localized to the upper left quadrant. However, she reported that the pain progressively worsened to a ten out of ten severity with a transition to sharp, stabbing pain. The patient also reported associated unintentional weight loss with loss of appetite. Notably, she had been started on mirtazapine one month prior. By this time, she had been on a dosing schedule of 15 mg by mouth at night for three weeks and had just started a week of 30 mg nightly.

**Figure 1:**
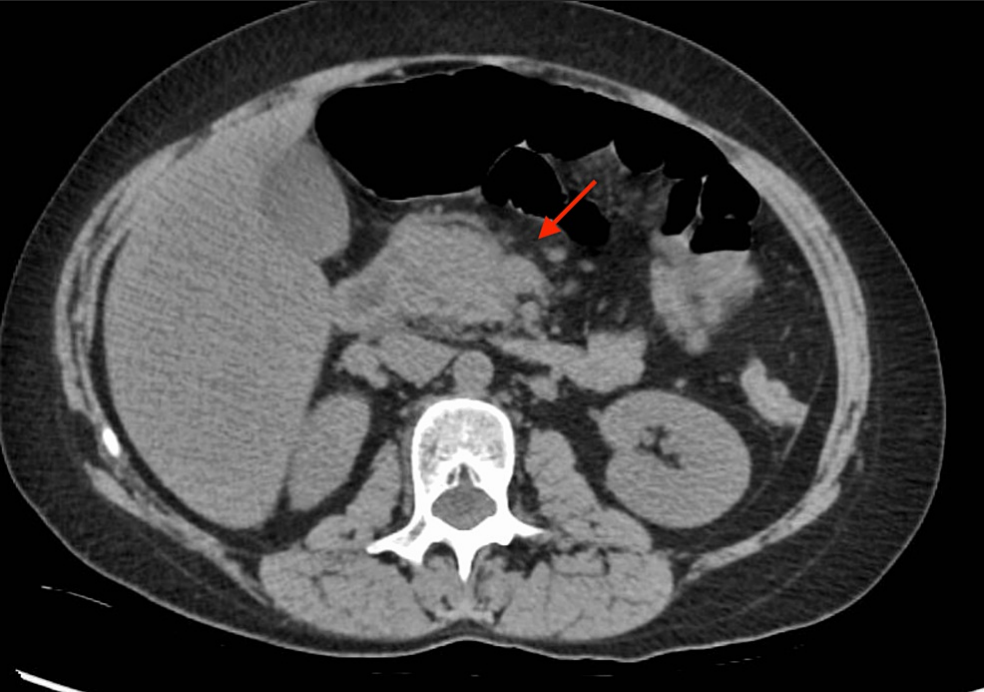
Transverse cross-section CT imaging of the abdomen with the arrow depicting edema surrounding the pancreas.

**Figure 2:**
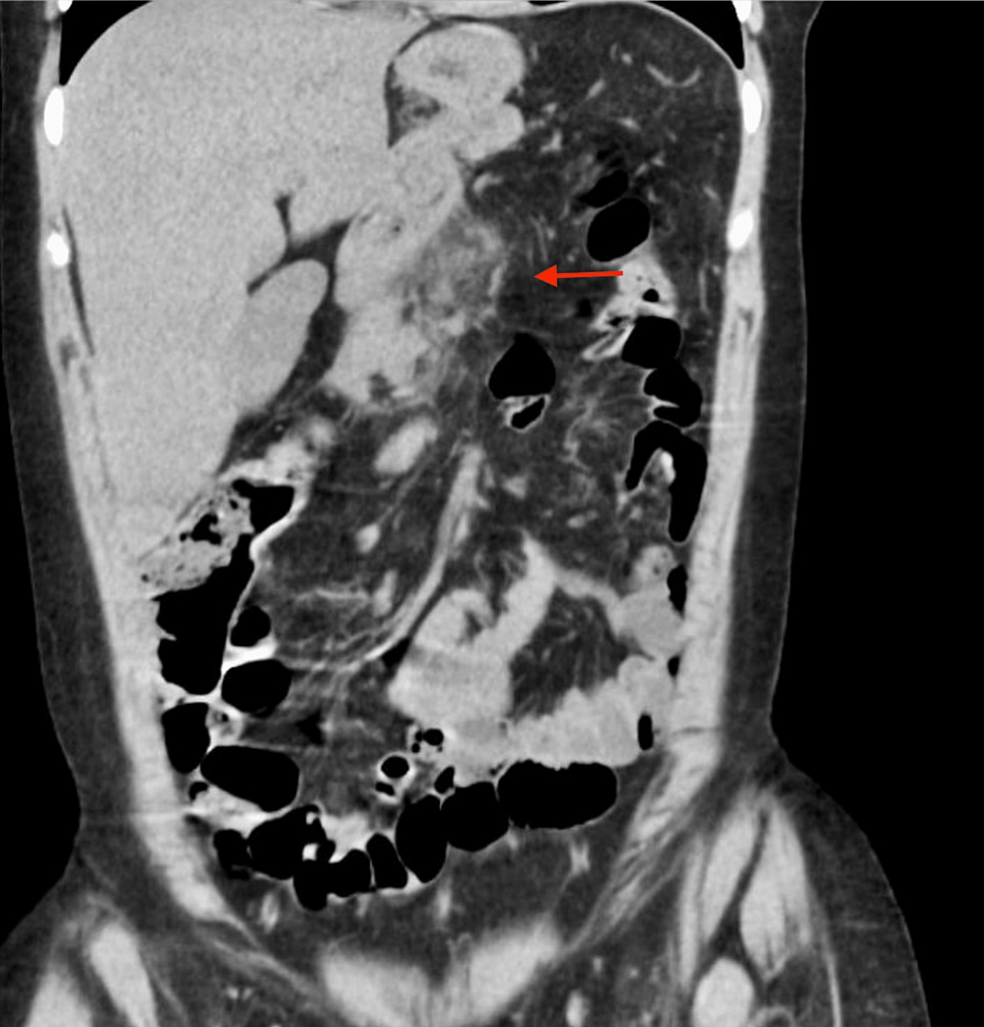
Vertical cross-section CT imaging of the abdomen with the arrow depicting edema surrounding the pancreas.

Further questioning revealed that the patient experienced nausea but denied vomiting. Physical examination showed severe epigastric pain on palpation. The patient’s vitals were unremarkable, except for sinus tachycardia evident on electrocardiography. Initial investigations indicated elevated lipase levels (700 U/L) and severe hypertriglyceridemia (>1,000 mg/dL). Complete blood count and complete metabolic panel were within normal limits. CT scan of the abdomen showed pancreatic edema, suggestive of AP (Figures 1, 2).

The patient was diagnosed with AP secondary to hypertriglyceridemia and was admitted to the hospital. She received appropriate pain control and intravenous fluids and was started on an insulin infusion with dextrose-containing fluids after consultation with gastroenterology. Mirtazapine was discontinued, as it was suspected to be the cause of elevated triglyceride levels. Unfortunately, the patient’s pain did not improve and required fentanyl.

Her triglyceride levels slowly began to downtrend, but to expedite the process, plasmapheresis was ultimately initiated. She underwent two cycles, with significant improvement in symptoms after the first. Her triglyceride levels continued to improve, and she was discharged home in stable condition with close outpatient follow-up.

## Discussion

AP is a very common gastrointestinal pathology [4]. It is most commonly associated with alcohol use disorder and gallstones; however, other etiologies have also been identified. These can include hypertriglyceridemia, medications, procedures such as endoscopic retrograde cholangiography, hypercalcemia, and trauma. AP can be diagnosed using the Atlanta criteria by meeting two out of the following three criteria: abdominal pain consistent with the disease, serum amylase and lipase three times the upper limit of normal, and/or characteristic findings on abdominal imaging.

Mirtazapine is a noradrenergic and specific serotonergic antidepressant that performs its function by blocking 5-HT2 and 5-HT3 receptors, as well as antagonizing alpha-2-autoreceptors and alpha-2-heteroreceptors. This mechanism of action leads to an increased release of norepinephrine and serotonergic transmission [5]. Mirtazapine is metabolized by cytochrome P450 isoenzymes in the liver [5]. It has been associated with weight gain and elevated triglyceride and cholesterol levels [6]. According to its manufacturers in a US-controlled study, non-fasting cholesterol levels were increased to greater than 20% above the upper limit of normal in 15% of patients. Additionally, non-fasting triglyceride was increased to greater than 500 mg/dL in 6% of patients treated with mirtazapine [6].

There have been limited documented occurrences of mirtazapine-induced hypertriglyceridemia leading to pancreatitis. One such instance was reported by Stone et al. [7] in which a young woman presented to the hospital with acute epigastric pain and was subsequently diagnosed with AP with triglyceride levels exceeding 4,800 mg/dL. The cause was attributed to mirtazapine and the medication was discontinued. The patient’s triglyceride levels showed a pattern of resolution within 36 hours, and follow-up visits indicated normal levels. Similarly, Bowers et al. [8] reported a case of an African American woman who presented with pancreatitis and elevated triglyceride levels that were linked to mirtazapine use. After discontinuation of the medication, the patient’s symptoms improved following insulin infusion, and the medication was no longer used. In a systematic analysis conducted by Milosavljevic et al. [3], nine cases of mirtazapine-induced pancreatitis were reviewed. In all cases, patients were hospitalized and treated for elevated triglyceride levels. Six of the nine cases underwent de-challenge, which was followed by improvement. Another instance, reported by Chen et al. [9], involved a 44-year-old woman with a history of depression who was diagnosed with AP two months after initiating mirtazapine therapy. She was found to have elevated serum triglyceride levels, along with amylase and lipase. The medication was stopped, and the patient’s symptoms quickly resolved with supportive care.

In previous case reports, it has been observed that discontinuation of mirtazapine results in the improvement of pancreatitis symptoms. Consistent with this trend, in our patient, discontinuation of mirtazapine led to improvement in symptoms and triglyceride levels. Before discontinuation, some studies utilized the Naranjo scale to assess the probability of an adverse drug reaction. This scale was initially developed to evaluate causality in adverse drug reactions during clinical trials, but it has also been applied in clinical practice. The Naranjo scale involves a series of questions and produces a score ranging from -4 to +13. A score of 0 or less is considered doubtful, 1 to 4 is possible, 5 to 8 is probable, and 9 or higher is definite [10]. Our patient scored +6 indicating that the reaction was probable. The Naranjo scale, however, has limited sensitivity and specificity in evaluating medication-induced hepatic injuries. Despite its limitations, the Naranjo scale is simpler to use compared to other methods for evaluating adverse drug reactions.

## Conclusions

Mirtazapine has been known to cause elevated triglyceride levels; however, the mechanism of action remains unclear. There have been a few proposed theories possibly through dysregulation of chylomicrons leading to elevated triglyceride levels and eventually causing pancreatitis. The second proposed mechanism refers to pre-existing underlying pancreatic injury caused by other etiologies which can lower the threshold for medication-induced pancreatitis. Although abdominal surgeries have been associated with pancreatitis, because her surgeries had been many years prior and her mirtazapine initiation preceded the acute event, we believe the etiology is more likely the latter. Considering the side effect profile of mirtazapine, clinicians should assess the risk versus benefit of initiating patients on medication for depression and should frequently monitor for elevated serum triglyceride levels and slow dosage increase to be introduced in patients with possible underlying pancreatic dysfunction.
